# Microtopographical cues promote peripheral nerve regeneration via transient mTORC2 activation

**DOI:** 10.1016/j.actbio.2017.07.031

**Published:** 2017-09-15

**Authors:** Suzanne E. Thomson, Chloe Charalambous, Carol-Anne Smith, Penelope M. Tsimbouri, Theophile Déjardin, Paul J. Kingham, Andrew M. Hart, Mathis O. Riehle

**Affiliations:** a The Centre for Cell Engineering, College of Medical, Veterinary and Life Sciences, University of Glasgow, University Avenue, Hillhead, Glasgow G12 8QQ, UK; b Canniesburn Plastic Surgery Unit, Glasgow Royal Infirmary, 84 Castle Street, Glasgow G4 0SF, UK; c Institut Jacques Monod CNRS UMR 7592, Université Paris Diderot, Paris, France; d Dept. of Integrative Medical Biology, Umeå University, SE-901 87 Umeå, Sweden

**Keywords:** Microtopography, Peripheral nerve, Tissue engineering, mTOR

## Abstract

Despite microsurgical repair, recovery of function following peripheral nerve injury is slow and often incomplete. Outcomes could be improved by an increased understanding of the molecular biology of regeneration and by translation of experimental bioengineering strategies. Topographical cues have been shown to be powerful regulators of the rate and directionality of neurite regeneration, and in this study we investigated the downstream molecular effects of linear micropatterned structures in an organotypic explant model. Linear topographical cues enhanced neurite outgrowth and our results demonstrated that the mTOR pathway is important in regulating these responses.

mTOR gene expression peaked between 48 and 72 h, coincident with the onset of rapid neurite outgrowth and glial migration, and correlated with neurite length at 48 h. mTOR protein was located to glia and in a punctate distribution along neurites. mTOR levels peaked at 72 h and were significantly increased by patterned topography (p < 0.05). Furthermore, the topographical cues could override pharmacological inhibition. Downstream phosphorylation assays and inhibition of mTORC1 using rapamycin highlighted mTORC2 as an important mediator, and more specific therapeutic target. Quantitative immunohistochemistry confirmed the presence of the mTORC2 component rictor at the regenerating front where it co-localised with F-actin and vinculin. Collectively, these results provide a deeper understanding of the mechanism of action of topography on neural regeneration, and support the incorporation of topographical patterning in combination with pharmacological mTORC2 potentiation within biomaterial constructs used to repair peripheral nerves.

**Statement of Significance:**

Peripheral nerve injury is common and functionally devastating. Despite microsurgical repair, healing is slow and incomplete, with lasting functional deficit. There is a clear need to translate bioengineering approaches and increase our knowledge of the molecular processes controlling nerve regeneration to improve the rate and success of healing. Topographical cues are powerful determinants of neurite outgrowth and represent a highly translatable engineering strategy. Here we demonstrate, for the first time, that microtopography potentiates neurite outgrowth via the mTOR pathway, with the mTORC2 subtype being of particular importance. These results give further evidence for the incorporation of microtopographical cues into peripheral nerve regeneration conduits and indicate that mTORC2 may be a suitable therapeutic target to potentiate nerve regeneration.

## Graphical abstract

**Figure:**
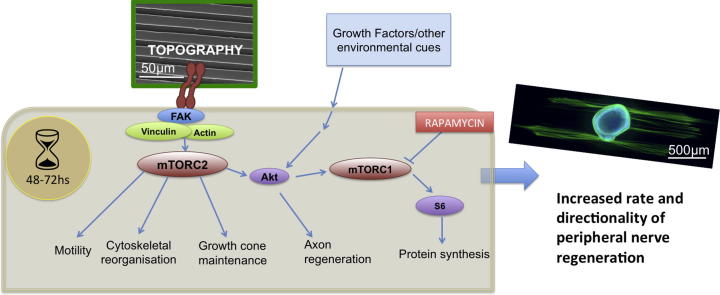

## 1 Introduction

Peripheral nerve injury is common secondary to trauma or oncological resection, is functionally devastating, and confers a significant socioeconomic cost [1]. Despite the innate regenerative capacity of the peripheral nervous system, recovery following injury remains slow and incomplete [2]. The extent of regeneration depends on the intrinsic growth capacity of the neurons, and environmental cues that regulate the temporospatial activation of inhibitory and pro-regenerative signalling cascades [3]. The current surgical gold standard for the treatment of a gap defect is timely reconstruction using an autologous nerve graft [4]. This provides cues in the form of natural anatomical internal topography, extracellular matrix, and Schwann and other support cell populations [5]. However, limitations include size mismatch, inadequate donor nerve length, sub-optimal internal topography, and donor site morbidity [6]. Currently available FDA/CE approved alternatives fail to surpass outcomes achieved using autologous nerve grafts [7].

### 1.1 Biophysical regulation of the nerve healing microenvironment

The ability of natural and engineered topographical cues to regulate cell phenotype and function is well described [8], [9], [10], [11], [12], [13], [14], [15], [16], [17], [18]. Directional control of peripheral nerve regeneration has been achieved in 2D and 3D, and is a key strategy in the design of a bioengineered nerve conduit [19]. Understanding the downstream mechanism of action of these pro-regenerative environmental cues informs biomaterial design and engineering, but also reveals mechanistic understanding relevant to identifying therapeutic biological targets for neuroprotection, or to enhance the rate and directionality of axonal regrowth across the site of repair and to the target organs.

### 1.2 The role of topography

Topographical cues alter cell shape and may act in concert with biochemical environmental cues. The mechanism of cellular response to topographical cues is cell and topography type specific and its detail have yet to be fully elucidated [9], [20]. However, given the ability to engineer topographies with nano- and micro-scale precision, an increased appreciation of these responses will have wide ranging implications in the design of the next generation nerve conduits [19].

Previous studies have identified that phosphoinositide 3-kinase/Akt/mammalian target of rapamycin (P13K/Akt/mTOR) signalling, mitogen-activated protein kinase (MAPK) signalling, and CrAT dependent pathways are of importance in neural regeneration. Carnitine O-acetyltransferase (CrAT) is an enzyme involved in mitochondrial regulation and is highly active in neuronal cells [21]. Two previously identified putative adjuvant pharmacotherapies are the CrAT substrates N-acetyl-cysteine and L-acetyl-carnitine [22], which enhance mitochondrial function and prevent neuronal cell death following injury [22], [23]. The mammalian target of rapamycin signalling pathway, mTOR, is a major regulator in neural regeneration and co-ordinates responses to growth factors, hormones, neurotransmitters and stress, making mTOR signalling a strong candidate for regulation of neuronal response to topographical cues.

mTOR is a highly conserved serine threonine kinase [24], [25] that exists in two distinct complexes mTOR complex 1 and mTOR complex 2 (mTORC1, mTORC2 respectively). mTORC1 is rapamycin sensitive and responsible for the regulation of cell growth, mRNA biogenesis and ribosomal translation [26]. mTORC2 contains a rictor complex in place of mTORC1’s raptor complex, and has been shown to regulate cytoskeletal remodelling in non-neuronal cell types [26].

Upstream regulation of mTOR by other environmental cues has been described [27], [28], [29], however it is not known if the neuronal response to topography is mediated by mTOR. Downstream phosphorylation of S6 and Akt(Ser473) are used as surrogate markers of mTORC1 and mTORC2 activity respectively [30]. This study aimed to investigate the relevant downstream molecular response of rodent primary sensory neurons to topographical cues. We hypothesised that mTOR is important in mediating this response. We performed phosphorylation assays and inhibited mTOR using rapamycin in order to investigate this. Rapamycin is a macrolide antifungal agent used to prevent rejection following transplantation [31]. *In vitro* and *in vivo* preclinical studies provide contrasting evidence as to the impact of rapamycin on nerve regeneration [32], [33], [34] and to date it has not been investigated by clinical trials in this context. Unravelling the regulatory mechanisms provides further characterisation of a useful *in vitro* model of nerve repair, and seeks future targets for therapeutic manipulation by pharmacology or bioengineering of nerve conduits [35], [36].

## 2 Materials and methods

### 2.1 Polymer fabrication, surface treatment and characterisation

To study the effect of topography *in vitro*, micro-patterned and flat polydimethylsiloxane (PDMS) constructs were cast. UV photolithography was used to pattern SU-8 photoresist on a silicone master wafer to be used as a template for fabrication of PDMS substrates. Pre-defined master wafer parameters (parallel grooves, 12.5 μm width × 5 μm height) were used, based on previous studies [37], [38]. The master wafer was centred on a casting jig and secured with screws. A PDMS mix (ratio of 10:1 base: curing agent, Silicone elastomer 184 SYLGARD®) was homogenised, degassed in a vacuum chamber for 20mins, injected into the casting jig, and cured for 2 h at 80 °C. Flat substrates were constructed using the same technique with an un-patterned master. The biocompatible, biodegradable polymer poly-ε-caprolactone (PCL) was also spin coated onto the surface of a SU-8 master generating identical micropatterns in a polymer suitable for future nerve conduit development. Air plasma treatment of the surfaces was used to increase hydrophilicity (Harrick Plasma PDC-002 Plasma Cleaner 29.6W, 1 min).

Following sterilisation by immersion in 70% ethanol for 30 min polymer surfaces were incubated in 13 μg/mL poly-l-lysine (PLL; MW ∼100,000, Sigma P1274) in reverse osmosis (RO) water for 30 min. Surfaces were then washed thrice with 1% PBS and blown dry with filtered (0.4 μm) air prior to use. Scanning electron microscopy (SEM) was performed with a Hitachi S800 field emission SEM at an accelerating voltage of 6 kVin SEM (high-vacuum) mode. Polymers were mounted on SEM sample holders and sputter-coated with gold prior to imaging to characterise substrate surfaces. Water contact angle was measured to confirm increased hydrophilicity following surface treatment using a KSV CAM 100 goniometer (KSV Instruments, Finland).

### 2.2 DRG organotypic explant

All work was carried out in strict accordance to the Home Office Animals (Scientific Procedures) Act 1986. Neonatal Sprague Dawley rats were euthanized by euthatal injection (Merial, 200 mg/ml, 500 mg/kg). Dorsal root ganglia (DRG) were harvested using careful microsurgical technique under binocular magnification. Explants were cultured at 37 °C, 5% CO_2_ in L-15 media (Sigma-Aldrich) supplemented with 10% FBS (Sigma-Aldrich), 50 μg/ml N-Acetyl-Cysteine (Sigma-Aldrich), 1% Penicillin-Streptomycin (GE Healthcare) and 10 ng/ml NGF 2.5S (Invitrogen). Rapamycin (Sigma-Aldrich) was reconstituted in dimethyl sulfoxide (DMSO, Sigma-Aldrich) and delivered daily in culture media to treated groups at 1 μM. An equivalent volume of DMSO (0.0036% v/v) was delivered in vehicle control groups. Media was changed every 24 h in all conditions. Standard incubator settings of 37 °C, 5% CO_2_ were maintained throughout the experiment.

### 2.3 Neurite outgrowth and migration assay

Neurite outgrowth and cell migration was measured at 2, 24, 48, 72 h, and at 5 and 10 days following explantation using 10x light microscopy (Motic AE31, Scion digital camera) and scanning fluorescence microscopy (Olympus, Axiovert). The outlines of the cluster of cell bodies within the ganglion and the extent of the emerging neurite front were defined and images analysed using Fiji software [39]. Based on the Herbert equation [40], total DRG explant area, maximal neurite length (defined as the furthest extension of a pathfinder neurite), and total surface area covered by neurites was quantified.

### 2.4 Quantitative real time PCR analysis

At the predefined time points DRGs were removed from culture and placed in 20 μl RNAlater® (Life Technologies, Paisley), then stored at −80 °C prior to processing. Immediately harvested DRGs were subject to the same process and used as a day 0 baseline control. Analysis of gene expression was performed using the 2^−ΔΔC^_T_ method [41] normalising expression to GAPDH and day 0 levels.

Total RNA from each time point was extracted using a Qiagen RNeasy micro kit according to the manufacturer’s protocol. DRGs were removed from cold storage and added to a solution of 350 µl of lysis buffer with β-mercaptoethanol plus 20 ng of carrier RNA (as described in the RNeasy Handbook, Qiagen) added. A triple disruption and homogenization technique was used, combining 30 s vortex, 5x passes with 26-gauge needle and 2 min centrifugation. An equal amount of RNA from each sample was used for cDNA preparation using the QuantiTect RT-PCR kit from Qiagen following the Qiagen protocol. qRT-PCR was carried out using the Quantifast SYBR Green kit (Qiagen) and a 7500 Real Time PCR system (Applied Biosystems). A minimum of 3 biological and 3 technical replicates was tested at each time point. The primer sequences (Table 1) for the genes were derived from Primer-BLAST [42] and validated by dissociation curve/melt curve analysis.

**Table 1:** Forward and reverse primer sequences used for polymerase chain reaction.

mTOR	FOR 5′ GCA GCA TGG GGT TTA GGT C – 3′
	REV 5′ CCC GAG GAA TCA TAC ACG TC – 3′
MAP3K12	FOR 5′ GTC TTC ACC TGC CTG TAC CC – 3′
	REV 5′ GGT TCC GAG GTT TGC TCT T – 3′
CrAT	FOR 5′ TAG CTT TTG CTC CCA GAA CC – 3′
	REV 5′ GGC CTT AAA TCG ACC AGA CA – 3′
GAPDH	FOR 5′ GGG TGT GAA CCA CGA GAA AT – 3′
	REV 5′ ACT GTG GTC ATG AGC CCT TC – 3′

### 2.5 Immunohistochemistry and protein localisation

Following specified time periods DRGs were fixed in 4% formaldehyde/PBS (1%) for one hour at 37 °C. DRGs were then permeabilised in a buffer solution (10.3 g sucrose, 0.292 g NaCl, 0.06 MgCl2, 0.476 g HEPES, 0.5 ml Triton X-100 per 100 ml PBS) for one hour at 4 °C. This solution was exchanged for a blocking solution (1%BSA/PBS) for one hour at 37 °C. A cocktail of primary antibodies was applied (1:100 mouse anti-beta3 tubulin (Santa Cruz), 1:100 goat anti-mTOR (Cell Signalling) OR 1:100 goat anti-S100 (Abcam), OR 1:100 mouse anti-vinculin (Sigma), 1:100 rabbit anti-rictor (Abcam), 1:50 fluorescein-phalloidin (Invitrogen) (diluted in 1%BSA/PBS) and DRGs were incubated overnight at 4 °C. S100 was used as a Schwann cell marker. Samples were washed thrice (PBS/0.5% Tween-20; 5 min per wash), prior to overnight incubation at 4 °C with secondary antibody cocktail (1:100 biotinylated anti-mouse antibodies, Vector Laboratories, and 1:300 donkey Texas red anti-goat, Invitrogen). Total incubation time was 18 h to allow penetration into the DRG tissue. Fluorescein Streptavidin 1:100 (Vector Laboratories) in 1% BSA/PBS was then added to the sample for 30 min at 4 °C and washed again. Samples were washed thrice, prior to being mounted with DAPI anti-fade medium (Vecta-Shield, Vector Laboratories, Peterborough UK) and viewed by fluorescence microscopy. Quantitative analysis was performed using FIJI software [39].

### 2.6 In-Cell western

For quantitative immunofluorescence measurement DRGs were fixed overnight and permeabilised for one hour, at 4 °C. Incubation times were optimised to enable staining of the cell somata throughout the body of the DRGs. Following blocking (Odyssey Blocking Buffer) for 2 h at room temperature samples were incubated overnight in primary antibody cocktail (mTOR, pmTOR Ser 2448, or Akt, pAkt Ser 473 (Cell Signalling) at 1:1000 concentration). Following 5 washes with PBS Tween (0.5%) DRGs were incubated for a further 12 h with species-specific secondary antibody or CellTag conjugated to IR-Dye of 700 or 800 nm emission wavelength. An Odyssey CA® Imaging System (Li-COR Biosciences) was used to quantify signal intensities. Settings were optimised and maintained throughout individual experimental runs. Signal intensities were normalised to CellTag or to each other for phosphorylation ratios [43], [44].

### 2.7 Statistical analysis

Statistical analysis was performed using GraphPad Prism version 7.0b (Graphpad Software). Results are presented as mean ± SEM from a minimum of 3 individual DRG explants. Student *t*-test or Mann-Whitney test (non-parametric) and repeated measure one-way analysis of variance (ANOVA) or Friedmans test (non-parametric), were used to determine statistical significance between data, Spearman correlation was calculated for co-localization and p < 0.05 was considered significant and standard reporting denoted by asterisks (p < 0.05 = ^∗^, p < 0.01^∗∗^, p < 0.001^∗∗∗^, p < 0.0001^∗∗∗∗^).

## 3 Results

### 3.1 DRG neurite outgrowth is regulated by topography

**Fig. 1:**
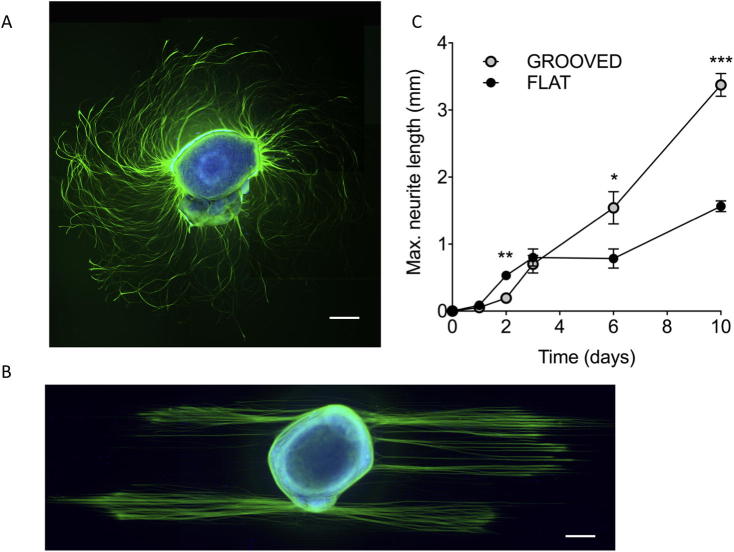
DRG explant cultured for 72 h on flat (A) or patterned (B) PLL coated PDMS substrates with 10 µg NGF/ml and stained for ß3-tubulin (green) and DNA (blue). A) Typical outgrowth on a flat substrate showing right turning spiral arrangement of axons. B) The topographical cues (12.5 μm wide, 5 µm deep) direct neurite outgrowth. Scale bars 200 μm. C) Plot of maximum neurite length versus time. Neurite length increases more rapidly on flat surfaces, until day 2, after which time topographical cues enhance neurite outgrowth (n ≥ 9, Mann-Whitney mean and SEM p < 0.05, ^**^p < 0.01).

For evaluation of neurite outgrowth in response to topographical cues micro-fabricated PDMS and PCL surfaces were produced with 12.5 µm wide grooves (Supplementary Fig. 5A), flat substrates were used as controls. Surface air plasma treatment rendered surfaces more hydrophilic (Supplementary Fig. 5B). The dorsal root ganglion (DRG) model was used for neurite outgrowth analysis, providing a 3D organotypic explant that more closely reflects the heterogeneous *in vivo* environment compared to dissociated neuronal cultures (Fig. 1A). A characteristic phenotypic response to topography was observed in the DRG explants (Fig. 1B). Topographical guidance cues resulted in highly directed neurite outgrowth, and significantly increased maximal neurite length from the third day in culture, compared to flat controls (Fig. 1C). Networks of DRGs grown on flat surfaces covered a greater surface area at day 3, with no difference in network area between substrates by day 10 (Supplementary Fig. 1).

### 3.2 Gene expression time course and effect of topography

**Fig. 2:**
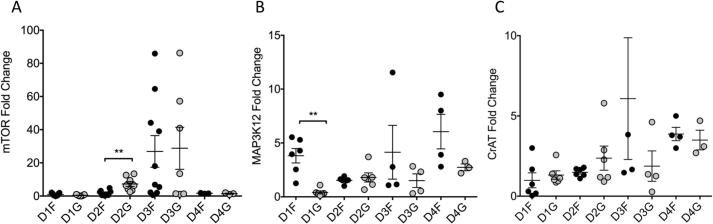
Timeline of candidate gene expression in DRG grown on grooved or flat substrates. Fold change presented normalized to day 0 control. All individual data points plotted alongside mean and SEM.A) mTOR: There was transient upregulation of mTOR on flat and grooved surfaces following injury. mTOR was significantly upregulated on grooved topography compared to flat substrates by day 2 (n ≥ 3, *t*-test, p = 0.004). Maximal upregulation was detected at day 3 with return to baseline by day 4. B) MAP3K12: Early increased transcription was demonstrated on the flat substrates (n ≥ 3, *t*-test, p = 0.002). Thereafter no statistically significant difference was seen between flat and grooved substrates. At all subsequent time-points there was increased expression on flat and grooved substrates compared to day 0 baseline. C) CrAT: upregulation from baseline was demonstrated on both flat and grooved substrates. No significant difference was detected between substrates, however, there is sustained upregulation over the duration of the study (Expression Day 1 vs. Day 4 Flat p = 0.0009, Grooved p = 0.03). One data point is an outlier (D3F = 17) and is off of scale.

Candidate gene expression was measured in each DRG sample and temporal regulation demonstrated (Fig. 2). mTOR gene expression increased to a peak at 72 h before returning to baseline and was significantly increased on grooved surfaces (p = 0.004) (Fig. 2A). We also investigated the impact on two other genes of known importance in nerve regeneration, MAP3K12 and CrAT. MAP3K12 gene expression showed less clear temporal variation and was upregulated on flat, but not grooved, substrates on day 1 (p = 0.002) (Fig. 2B). CrAT expression was increased over the time-course studied and there was no impact of topography upon CrAT expression (Fig. 2C).

### 3.3 Transient mTOR gene expression coincides with the period of rapid neurite outgrowth

**Fig. 3:**
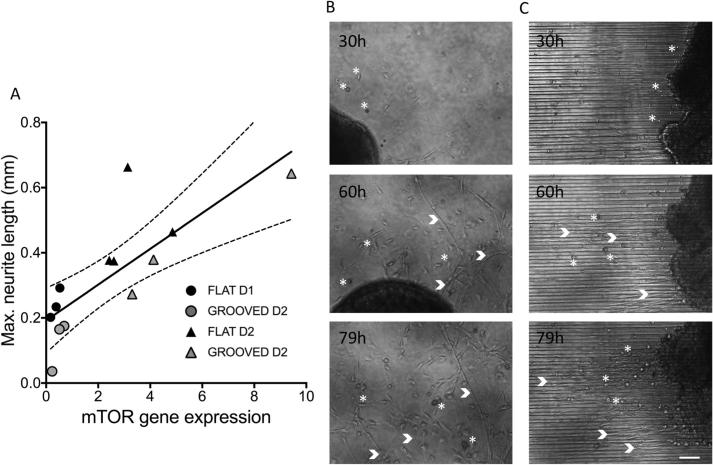
A) Timeline of mTOR activation, neurite extension and support cell activity of regenerating DRG neurons cultured with 10 ng/ml NGF. mTOR levels correlate well with neurite length in the first 2 days (Spearman correlation, r = 0.7920, 95% confidence intervals, p < 0.01, n = 13). The neurite length was measured for a subset of DRGs from Fig. 3A. Each point represents an individual DRG, day 1 (●), day 2 (□). Columns B, C) Time-lapse images of DRG regeneration on flat (C) and grooved substrates (D). Support cells (examples indicated by *) and axonal projections (>) and are captured, with increased activity demonstrated at 60hours. On the flat surface (C) axonal outgrowth and support cell movement is random. Highly directed neurite outgrowth and support cell movement on 12.5um linear topography (C). Supplementary movies available to online. Scale bar 100 μm.

As the greatest up regulation was detected in the mTOR transcripts we subsequently investigated this pathway in more detail over a ten-day timeline. By day two mTOR expression was upregulated on both flat and grooved substrates and mTOR gene expression correlated with neurite length (Fig. 2A Spearman rank correlation, r = 0.7920, p = 0.0012, n ≥ 12). Transcript levels were significantly greater on grooved substrates (n ≥ 3, *t*-test, p = 0.004). Maximal expression occurred 3 days following injury, and was coincident with the onset of a period of rapid neurite outgrowth and support cell activity (Fig. 3B, C). Time-lapse videos demonstrating outgrowth are available within supplementary files.

### 3.4 mTOR is upregulated in response to topography in both neurites and support cells

**Fig. 4:**
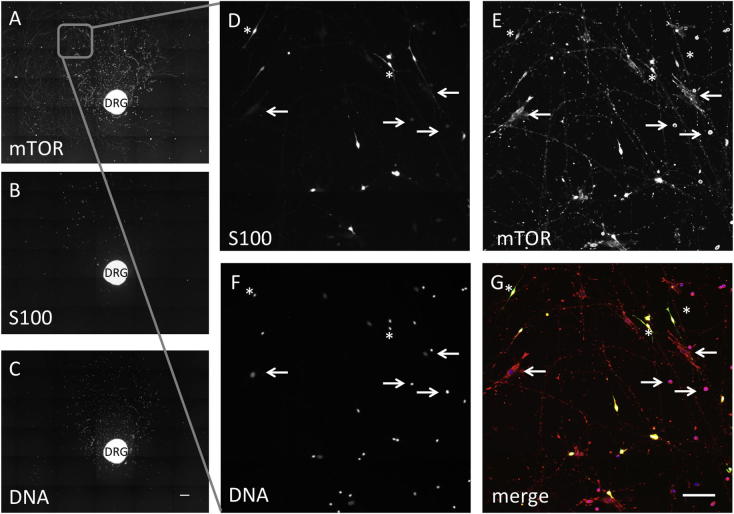
DRG cultured on flat substrates stained for mTOR, S100 and DNA. A) mTOR protein was located within the portion of the DRG explant containing neural cell bodies and in a punctate manner along regenerating neurites B) S100 positive cells, C) cell nuclei. Magnification of the area indicated in A in D-G. D) S100 positive cells. Arrows indicate the same S100 negative, mTOR (E) positive cells. E) mTOR positive cells. mTOR was detected within distinct S100 positive (*) and negative (arrows) support cell subpopulations in D and E. F) DNA image showing all cells. G) Merge of mTOR (red), S100 (green) and DNA (blue) images. Further analysis identified a subpopulation of the mTOR+/S100- cells as ED1 positive macrophages (data not shown). Scale bar 400 μm (A-C), 100 μm (D-G).

**Fig. 5:**
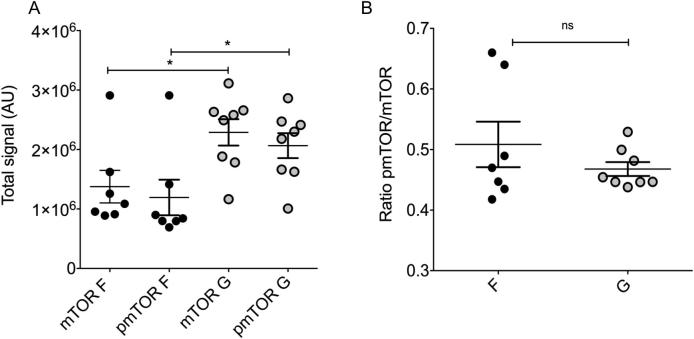
mTOR phosphorylation on flat (F) and grooved (G) substrates following 72 h in culture (NGF 10 ng/ml). A) mTOR and phosphorylated (pmTOR) protein were significantly increased in DRGs grown on grooved topography (*t*-test, mTOR p = 0.02, pmTOR p = p = 0.03). B) Ratio of pmTOR/mTOR plotted for flat and grooved samples at 72 hs shows that there was no difference in the ratio of phosphorylated mTOR in either condition.

mTOR protein staining was located in a punctate manner along outgrowing neurites and also within distinct S100 positive (Schwann/satellite cells) and negative support cell subpopulations. Staining was demonstrated throughout the neuronal perikarya and support cells in the ganglia (Fig. 4). Li-COR In-Cell Western (Fig. 5) was used to quantify total and active (phosphorylated) mTOR protein and normalised to CellTag (unspecific protein stain used for normalisation, Li-COR) [43]. At the 72-h time point total mTOR expression was increased on grooved topography compared to flat surfaces (p = 0.02), as was the total active phosphorylated protein (p = 0.03). These results confirmed that the detected changes in gene expression correlated with increased protein translation.

### 3.5 Topographical cues produce differential responses to rapamycin

The mTOR pathway was further interrogated using the pharmacological inhibitor rapamycin. The activation of downstream pathways was studied to identify critical elements in the response to topography - here phosphorylation of S6 and Akt (Ser473) were used to monitor mTORC1 and mTORC2 activity respectively [30].

**Fig. 6:**
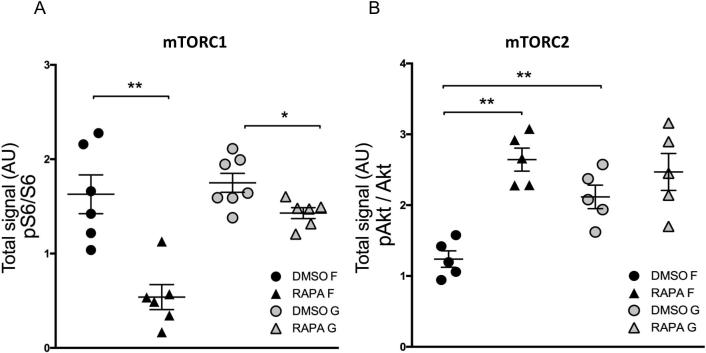
Impact of rapamycin treatment after 48 h of culture on S6 ribosomal protein and Akt if cultured on flat or grooved substrate. DRG were maintained with 10 ng NGF/ml. A) Ratio of phosphorylated S6 ribosomal protein (pS6) to all S6 ribosomal protein. Pharmacological inhibition of mTOR by rapamycin was confirmed by significant reduction in ratio of phosphorylated S6. Despite similar baseline ratios a greater reduction of S6 activity in response to rapamycin treatment was seen in DRGs cultured on flat substrates (Mann-Whitney, p = 0.0043, n = 5). B) Ratio of phosphorylated Akt over total Akt in relation to rapamycin treatment. Baseline pAkt ratios (indicating mTORC2 activity) were significantly greater in DRGs cultured on topography. Inhibition of mTORC1 by rapamycin resulted in increased mTORC2 activity on flat, but not on grooved substrate.

Pharmacological inhibition of mTOR by rapamycin was confirmed by significant reduction in pS6 levels (Fig. 6A), an effect that was potentiated by culture on flat surfaces (despite comparable initial baseline levels on both substrates). Use of grooved substrates appeared to decouple the link between mTORC1 and mTORC2 levels. When compared to flat surfaces, grooved topography supported increased baseline Akt phosphorylation (mTORC2 activity marker), and provided resistance against the effect of mTORC1 inhibition by rapamycin (Fig. 6B).

**Fig. 7:**
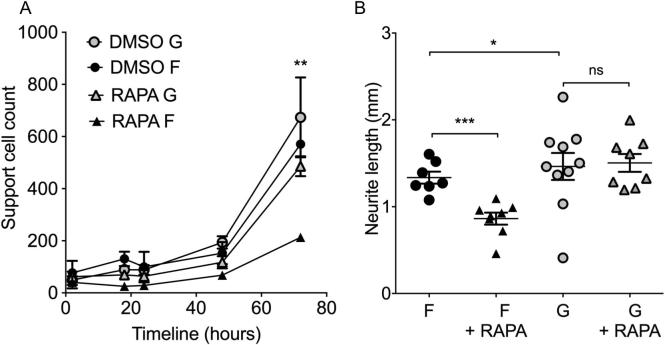
Support cell number (A) and neurite outgrowth (B) in response to rapamycin treatment. 1 µM rapamycin consistently reduced the number of support cells on flat surfaces (F) compared to grooved surfaces (G) (P = 0.0021 Friedman test – non-parametric equivalent of one-way ANOVA), this was particularly evident by 72 h (*t*-test, with Welch’s correction, RAPA F vs. RAPA G at 72 h, p = 0.0003, N > 7). There is a trend toward increased support cell number on grooved surfaces (grey symbols) compared to their flat counterparts (black symbols). Grooved surfaces consistently increase neurite outgrowth compared to flat surfaces (p = 0.005 for NGF, p = 0.285 for DMSO, p = 0.028 for RAPA). (72 h). Rapamycin treatment significantly reduced neurite outgrowth on flat surfaces (▴) compared to DMSO controls (●) (*t*-test, with Welch’s correction, p = 0.0003), but grooves did not show this phenomenon.

A differential response to rapamycin treatment was consistently observed on grooved vs. flat substrates. Rapamycin treatment reduced the number of support cells (Fig. 7A) and maximal neurite length (Fig. 7B) on flat surfaces, but had no effect on grooves. Taken together these results demonstrate a differential regulation of the mTORC1/S6 and mTORC2/Akt pathways by topographical cues.

### 3.6 Localisation of mTORC2 component rictor at the regenerating front

**Fig. 8:**
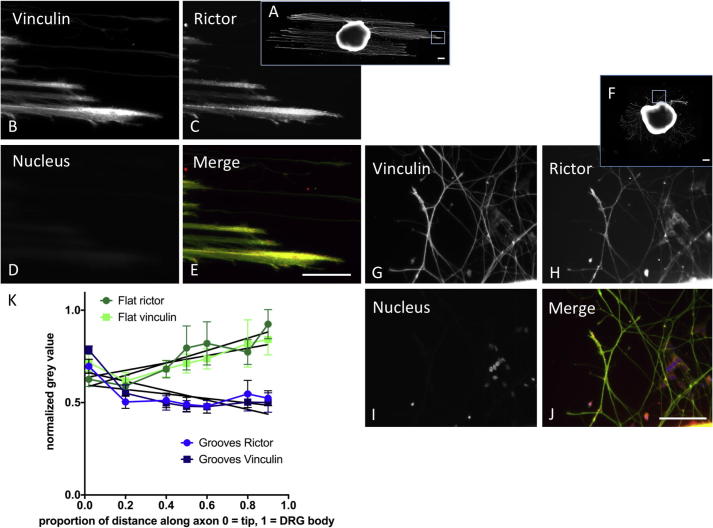
DRG cultured on topographically patterned (A-G) and flat (F-I) substrates stained for vinculin (A, B, F, G), rictor (mTORC2) (C, H) and DNA (D, I). In the merged images E and J) Rictor (red) and vinculin (green) co-localize at growth cones (yellow), with maximal intensities close to the leading edge of the growth cones (yellow). Scale bar: 100 μm in images E and J applies to B-E and G-J respectively; 200 μm in whole DRG images A and F. K) Plot of normalized grey value of rictor (●) and vinculin (■) against relative distance along axon, from the tip (0) towards the DRG body (1). Values for flat surfaces are in green, topographically patterned in blue. Rictor and vinculin co-localize at the regenerating tip in DRGs cultured on both substrates and the normalized grey values demonstrate correlation (Spearman correlation between vinculin and rictor for grooves r = 0.79, p < 0.05, flat r = 0.75, p = 0.063, n = 36).

**Fig. 9:**
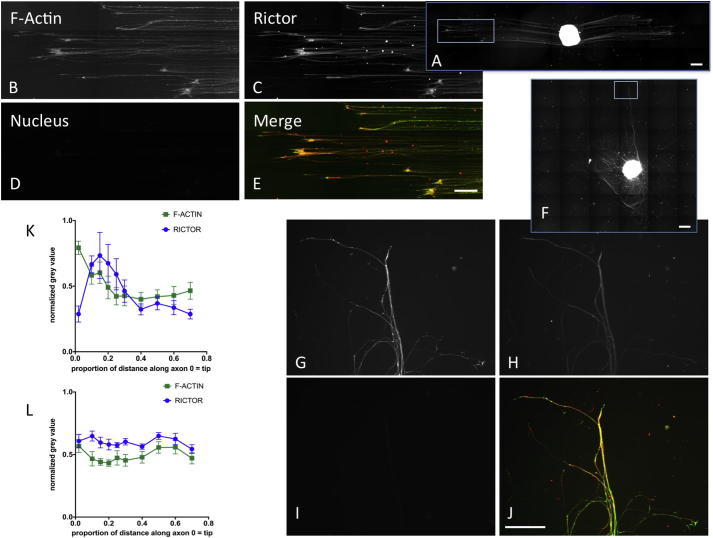
A) DRG cultured on topographically patterned and flat substrates stained for A, B, F, G) F-actin, C, H) rictor and D, I) DNA. E) Rictor (red) and F-actin (green) staining seen throughout neurites, with maximal co-localization at growth cones (yellow). Scale bar 200 μm in magnified images, 500 μm in whole DRG image. K) Plot of normalized grey value against distance along axon from tip towards cell body in DRGs cultured on topography (K) and flat substrates (L). Quantitative analysis demonstrates peak F-actin grey value at the tip of the growth cone, peak rictor grey scale is localized just proximal to this however relative intensity change along the neurite is not as striking as that seen on grooved surfaces (mean and SEM, n = 12). Grey values normalized to the maximum detected along the whole neurite.

**Supplementary Figure 2:**
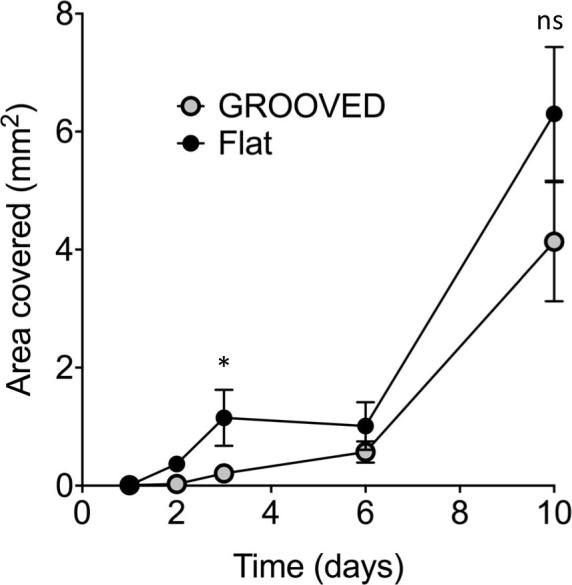
Plot of neurite network area versus time. Network area is greater on flat surfaces at day 3 (n ≥ 9, Mann-Whitney mean and SEM p < 0.05), after which time there is no difference between substrates.

**Supplementary Figure 3:**
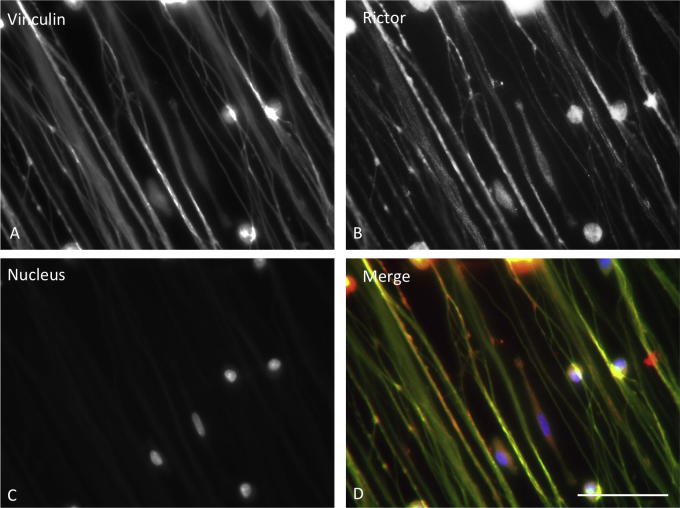
Magnified image of neurites and support cells growing from a DRG cultured on topographically patterned substrate stained for vinculin, rictor and DNA. Rictor (red) staining seen in a punctate manner throughout neurites and within support cells. Scale bar 100 μm.

**Supplementary Figure 4:**
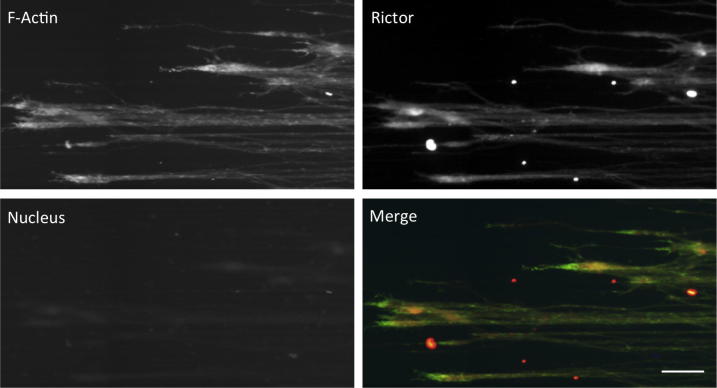
Neurites growing from a DRG cultured on topographically patterned substrate stained for F-actin, rictor and DNA. Maximal intensity of F-actin (green) and rictor (red) staining at growth cones. Scale bar 100 μm

**Supplementary Figure 5:**
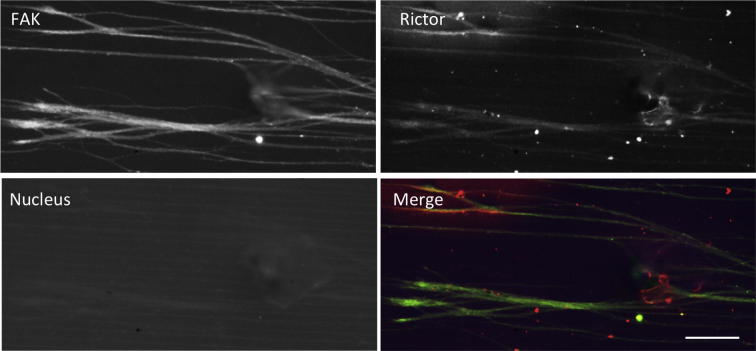
Neurites growing from a DRG cultured on topographically patterned substrate stained for FAK, rictor and DNA. Maximal intensity of rictor (red) staining at regenerating growth cones. Scale bar 100 μm

To investigate the hypothesis that mTORC2 is important at the regenerating front we stained for rictor, vinculin, F-actin and focal adhesion kinase (FAK) (Fig. 8, Fig. 9 and Supplementary Figs. 2, 3, 4). We found that vinculin and rictor co-localised at growth cones (Fig. 8), irrespective of topography in all neurites. Quantitative analysis demonstrated close correlation in normalized grey values of rictor and vinculin on flat and grooved substrates (Fig. 8). The distribution along neurites was differentially regulated by topography. The tip of the growth cone was identified by peak F-actin grey value and an absence of rictor, with peak rictor grey scale localised just proximal to this point (Fig. 9). In DRGs stained for rictor and vinculin, but not actin (Fig. 8) it is likely that the tip of the growth cone was not detected, instead rictor and vinculin peak normalised grey value occurred at the same point.

## 4 Discussion

Improving the rate and directionality of nerve regeneration is a significant challenge. A combinatorial approach employing microsurgical and bioengineering strategies is required. This study demonstrates that linear topographical cues influence the network shape and directionality of DRG axons, as well as the time course of gene expression.

Our findings concur with previous reports that linear topographical cues, whether in the form of microgrooves (as in the current study), fibres, or channels enhance neurite outgrowth [45], [46], [47], [48], [49], [50] and we now provide new detail on the molecular response. Previous studies demonstrate DRG neurons respond to a range of linear guidance cues (width 1–100 μm) [51], [52], [53]. Anisotropic linear cues (12.5 μm in width and 5 μm in depth) and the organotypic explant model were used in this study. This facilitated the study of the response and interaction of several cell types to topographical cues, as is more representative of the *in vivo* environment. Schwann cells aligned to the topography, in keeping with previous findings [46], [54], and in addition we found that topography increased the migration and number of support cells. This would likely cause directional deposition of extracellular matrix molecules and could lead to haptotactic cues that are known to enhance directional axon outgrowth [55], [56].

### 4.1 Topography modulated expression of candidate genes

Understanding the downstream molecular responses to topography informs the design of future bioengineered conduits. We investigated three selected candidate genes of known importance in nerve regeneration, with rat:human homology and the potential for pharmacological modulation. The highly conserved MAPK pathway is of therapeutic importance since it is required for successful regeneration [57], [58]. MAP3K12 has several contrasting roles and knowledge of its temporospatial activity remains incomplete. It has been demonstrated that despite acting upstream of C-Jun terminal kinase (JNK) as a pro-regenerative switch, a reduction in MAP3K12 protects neurons from apoptosis after nerve injury and prevents Wallerian degeneration of distal axons in rodent models [59], [60], [61], [62]. In this model an early increase in MAP3K12 expression was found, with significantly raised transcript levels detected in DRGs cultured on flat substrates. However, despite continual expression MAP3K12 transcript levels were consistently reduced on topography, as opposed to flat, substrates. This indicates that topography may act to reduce activity of the MAP3K12 pathway and warrants further investigation.

Upregulation of the carnitine acetyl transferase (CrAT) pathway confers neuroprotection following injury [22], [63]. In keeping with the increased metabolic demand associated with growth, CrAT expression was consistently elevated in DRGs cultured in both conditions, and levels increased temporally (Fig. 2C) [64], [65].

The most profound transcript up regulation was observed in mTOR, and hence became the focus of this study.

### 4.2 mTOR is of critical importance within the first 72 h following injury

A large body of literature highlights the importance of the mTOR pathway in nerve injury and repair, and describes upstream regulation of mTOR by stress, growth factors, hormones and neurotransmitters [27], [33], [66], [67], [68]. However, the role of mTOR in mediating the response of regenerating neurons to topography and whether mTOR plays a role locally at the injury site, or distantly in the cell body had not been fully ascertained [67], [68]. This study provides new evidence upon the pattern of change in mTOR expression within the perikarya and growth cones, and confirms mTOR as an important downstream mediator of topographical cues. A consistent, transient, differential response in regeneration-related gene and protein levels was demonstrated between DRG cultured upon linear topographies and flat control surfaces.

Intrinsic mTOR levels decline with age and are correlated with decreased growth capacity in retinal ganglion cells [28]. Conversely, deletion of upstream mTOR inhibitors result in increased neural regeneration [27], [33]. This study demonstrates that linear microtopographical cues temporarily increased basal mTOR levels, peaking on day 3. These findings are in agreement with Abe et al. [33] who demonstrated transient activation of mTOR in DRG immediately after injury, with return to basal levels four days following injury. Love et al. evaluated axonal mTORC1 levels *in vivo* at early time points, and detected an increase at six hours following injury, attributed to local translation of protein transcripts in the axon [68]. Our time course and localisation detail indicates that mTOR is of importance in the initiation of outgrowth.

### 4.3 Topographical cues increase mTORC2/pAkt activity, independently of mTORC1

An earlier and increased up-regulation of total mTOR transcript and protein levels was detected when DRGs were cultured on grooved substrates. As mTOR exists in two different complexes, mTORC1 and mTORC2, their activity was monitored via phosphorylation of S6 and Akt(Ser473) respectively [30]. Baseline pAkt (Ser 473), a read out for mTORC2 [69], was shown to be increased in DRGs cultured on linear microtopography (Fig. 6) and was associated with increased neurite regeneration.

Pharmacological inhibition of mTORC1 reduced support cell proliferation, migration and neurite elongation on flat surfaces, however interestingly rapamycin had no impact on regeneration along linear topographical cues. Taken together these findings indicate that mTORC2 is more important in mediating the response to linear topography, whereas regeneration on flat surfaces is more reliant on intact mTORC1 activity.

Although much is known about how external environmental cues (e.g. nutrients, growth factors, energy and stress) regulate mTORC1, surprisingly fewer papers concern the response of mTORC2 to these stimuli [70].

Downstream of mTORC2, the pathway response involves RAC, Rho [71], [72], [73] PKC-α and SGK1. In retinal epithelia and CNS neurons rho GTPases and protein kinase C, both regulated by mTORC2, have been demonstrated to be of importance in mediating the response to micropatterning [74], [75]. Further studies of the roles of these pathways are necessary to delineate their precise roles in peripheral nerve axon guidance and growth cone dynamics [76].

Akt is of known importance in regulating growth and proliferation, by mediating many of the cellular effects of PI3K [77], [78]. However, many of the effects of Akt on other processes (e.g. migration) appear to be independent of PI3K [79]. mTORC2 is necessary for neuronal protrusion in *C. elegans* and in this model system cdc42 is an important mediator, acting upstream of PI3K, and known to be more critical than mTORC1 [76]. This study supports these findings, and furthermore indicates that mTORC2 is transiently involved in coordinating neuronal response to topographical cues.

A possible mechanism through which mTORC2 could perform this action would be through interactions at point contacts, which are similar to focal adhesions. Point contacts are involved in axon path finding and organised through membrane associated proteins, which are phosphorylated at tyrosine residues [80], [81]. It is logical that these could be initiated through topography as grooves have previously been demonstrated to initiate tyrosine phosphorylation of membrane proteins [82], [83].

### 4.4 Focal adhesions

Focal adhesions (FAs) are multifunctional protein agglomerates that mediate cell–ECM adhesion, force transmission, cytoskeletal regulation and signalling [83], [84] and form along physical features such as groove/ridge transitions [82]. As such, topographical cues present areas for FA formation to be initiated, causing cytoskeletal rearrangement, and can in other cell types act through both direct and indirect mechanotransduction to alter gene transcription [12], [82]. Although much is known about focal adhesion regulation in non-neuronal cells it is recognised that a more detailed molecular insight is required into how mechanical signals, encountered by the growth cone, initiate signalling [85], [86], [87]. Schulte et al. showed how the cellular interaction with the nanotopographical features can promote neuronal differentiative behavior in PC12 cells and hippocampal neurons [86], [87]. This is realised by the impact of the neuron/nanotopography interaction on mechanotransductive processes, such as FA maturation (restriction to point contact dimensions), cytoskeletal organisation and mechanics (i.e. stress fibre formation and cell rigidity). This has the net effect of polarisation, growth cone stabilisation and potentiated neuritogenesis. Furthermore, it was demonstrated that the response of neuronal cells to topography resulted in increased RhoA and cdc42, independent of NGF and TrKA. Both RhoA and cdc42 are downstream of mTORC2 and are known to activate FAK [86]. Our finding that mTORC2 is important in mediating the response to topography provides further molecular explanation, extending their results. In other cell types, predominantly stem cells, it has been demonstrated that mTORC2 co-localises with Akt at newly formed FAs, and becomes associated with vinculin when cells are exposed to strain to mediate cytoskeletal reorganization, independent of PI3K [79], [88]. Yang et al. demonstrated that a hierarchical patterned substrate influenced the fate of neural stem cells [89]. The co-localisation of FAK and vinculin supports the importance of FAs in mediating the differentiative process, which was reduced by the addition of integrin antibodies and ROCK inhibition [89]. Using co-localisation we demonstrated that mTOR was present in a punctate manner along neurites and at the regenerating front, which may represent mTORC2 activity at FAs. In order to test this hypothesis we investigated co-localisation of focal contact molecules FAK, F-actin and vinculin with rictor, the main component of mTORC2. We found that rictor co-localised with vinculin, F-actin and FAK on topography (Fig. 8, Fig. 9, Supplementary Figs. 2, 3, 4). The grey value of vinculin and rictor staining was greatest at the regenerating front, an effect that was differentially regulated by topography. F-actin was located at the very tips of exploratory filopodia and rictor co-localised at these points, with peak grey value just proximal to that of F-actin. Taken in context our data indicates that canonical mTORC2 signalling occurs within the regenerating front, at the growth cone. We propose that linear topographical cues act to increase mTORC2 activity, resulting in the detected phosphorylation of Akt, both through and independently of mTORC1/PI3K.

There are discrepancies found in the literature regarding the effect of rapamycin treatment on neuronal regeneration [32], [90], [91]. A detailed review reveals that in cases where topographic guidance was provided (e.g. natural or synthetic fibres/channels or surface topography), rapamycin increased or had no impact on neurite outgrowth [32], [90], [91]. On the other hand, where there were no surface features nerve regeneration was reduced by rapamycin [33], [92], [93]. The differential effect observed by addition of rapamycin to DRGs cultured on flat or patterned substrates in this study provides an explanation for these discrepancies. In light of these findings rapamycin has the potential to have a pro-regenerative impact, through well-recognised immunomodulatory effects, if delivered as part of a topographically patterned nerve conduit.

## 5 Conclusion

Although the mTOR pathway has previously been demonstrated to regulate neurite response to other environmental factors [26], [31], [94], [95], its role in mediating response to topography in dorsal root ganglia had not previously been described; furthermore the role of mTORC2 has been relatively overlooked. This study implicates mTORC2 as an important downstream mediator of topography in peripheral nerve regeneration. These findings support the incorporation of linear topographical cues into nerve repair constructs, and highlights the additional therapeutic potential if mTORC2/pAkt potentiating agents can be incorporated into the constructs.

## Figures and Tables

**Supplementary Figure 6:**
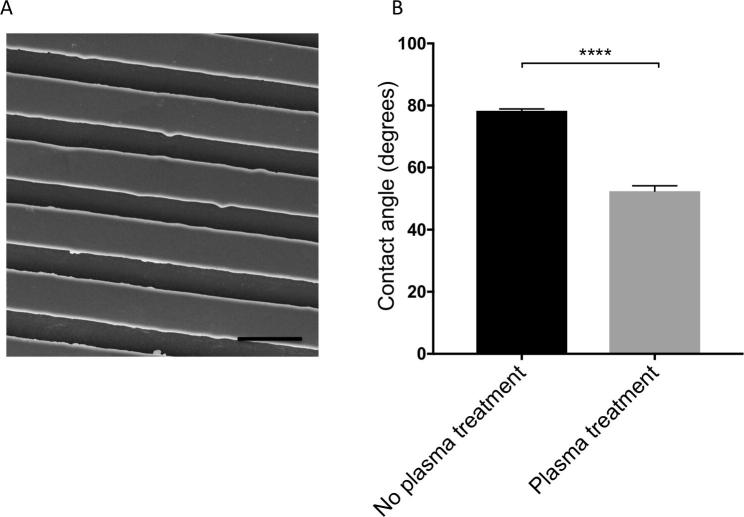
Characterization of PDMS. A) SEM demonstrates anisotropic topography 12.5 μm wide, 5 μm deep. Scale bar 20 μm. B) Substrate water contact angle of is reduced by air plasma treatment (mean of untreated surface = 79°, mean of treated surface 50°, n = 4 Mann Whitney test p < 0.0001)
