# Supplementary figures and images for: Microtopographical cues promote peripheral nerve regeneration via transient mTORC2 activation
Source: Acta Biomater. 2017 Sep 15;60:220–31. doi: 10.1016/j.actbio.2017.07.031 (PMC5593812; doi:10.1016/j.actbio.2017.07.031)

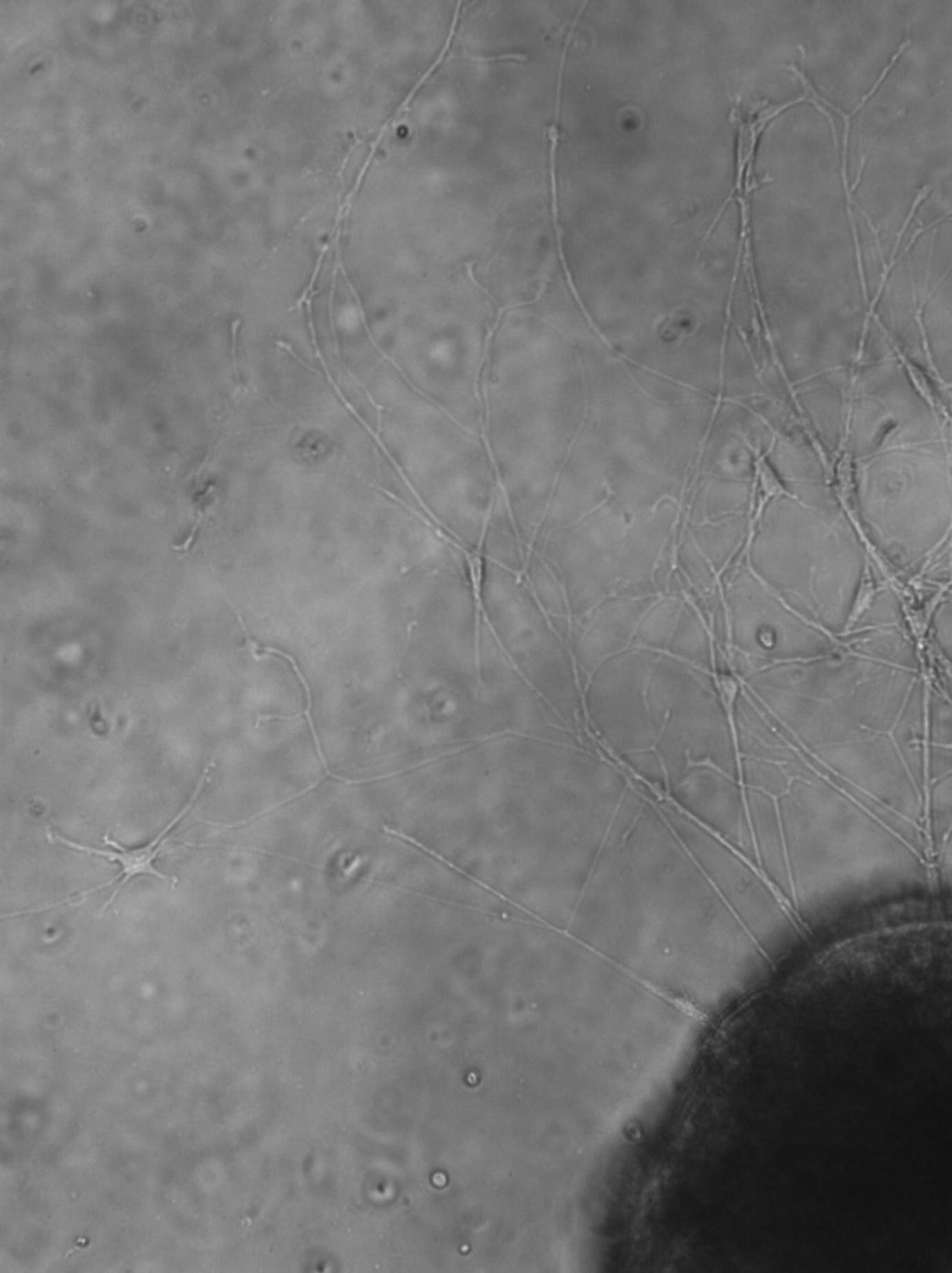

Supplement: Supplementary video 1 [file mmc1.jpg]

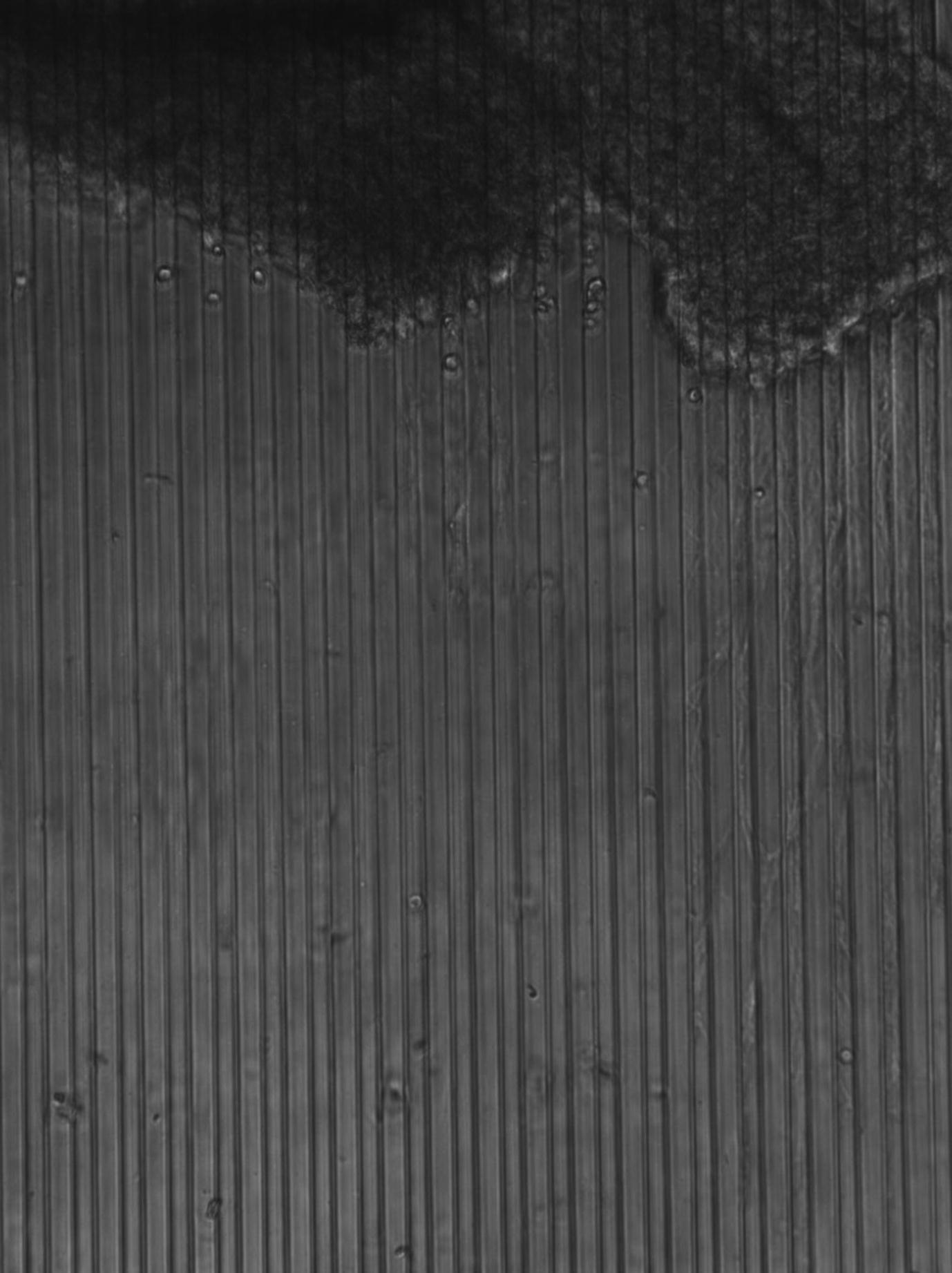

Supplement: Supplementary video 2 [file mmc2.jpg]
